# Factors associated with relief from acute pain among patients admitted in medical ward of Mbarara Regional Referral Hospital, south western Uganda: A cross-sectional study

**DOI:** 10.1371/journal.pone.0317919

**Published:** 2025-03-03

**Authors:** Silas Ojuka, Robert Tamukong, Tadele Mekuriya Yadesa

**Affiliations:** 1 Department of Pharmacy, Faculty of Medicine, Mbarara University of Science and Technology, Mbarara, Uganda; 2 Department of Clinical pharmacy and Pharmacy practice, School of Pharmacy, Kampala International University, Ishaka, Uganda; Makerere University College of Natural Sciences, UGANDA

## Abstract

Acute pain is an understudied subject among patients admitted in medical wards, especially in sub-Saharan Africa. Given that it is one of the commonest causes of hospital admissions, it is necessary to diagnose and adequately treat it in time. Unrelieved acute pain may have negative consequences such as; reduced quality of life, prolonged hospital stays and increased cost of treatment. The purpose of this study was to assess relief of acute pain and factors associated with it in medical ward of Mbarara Regional Referral Hospital, South-Western Uganda. Severity of pain was determined using the Brief Pain Inventory. Adequate drug therapy for acute pain was assessed using the Pain Management Index. Relief from acute pain was considered a change in pain grade from severe to mild or moderate to mild or mild to no pain. This was done by comparing baseline pain grade at enrollment (day one) and follow up pain grade on day two. Multivariate logistic regression was performed to identify associated factors that had statistical significance. Out of 280 patients with acute pain, analgesic drug therapy was adequate for 32 (11.43%) participants while relief from acute pain was achieved among 95 (34%). Multivariate logistic regression showed female gender to be significantly associated with relief from acute pain (adjusted Odds Ratio=1.86; 1.11-3.10 at 95% C.I; p value=0.018). Prevalence of adequacy of analgesic drug therapy for acute pain among patients admitted in medical ward of Mbarara Regional Referral Hospital was low. Proportion of patients with relief from acute pain was also low. Female patients were more likely to experience relief from acute pain compared to their male counterparts.

## Introduction

Over 80% of individuals seek health care only after experiencing pain [1] however it is important to note that the inability of an individual to verbally communicate the severity and nature of their pain does not mean they are not in pain. This is because pain is a highly subjective experience [2]. In spite of being protective to the body and aiding in survival, it is necessary to manage acute pain as it can cause harm if poorly controlled. Acute pain is described as pain of sudden onset with duration of not more than 12weeks [3]. Chronic pain on the other hand is of gradual onset and is associated with movement impairment for more than 12 weeks [4]. Unlike acute pain which is only a symptom of underlying injury chronic pain is a disease requiring more complex medical therapy [5].

The negative impact of pain is worse among the older patients compared to the younger ones [6]. Poorly treated pain may impair physical activity, cause depression and poor quality of life for the patient [7]. Severe levels of pain may cause patients to get distressed which negatively affects patient prognosis[8]. It is also associated with elevated levels of endogenous catecholamines which is associated with cardiovascular diseases like cardiac ischemia, increased risk of coagulation, thromboembolic disorders, poor glycemic control[9].

Acute pain has been highly studied in the western prehospital and hospital settings unlike in Africa [10]. Worldwide, approximately 1 in 5 adults experience pain [11] and yet it is estimated that 80% of the world’s population has limited access to treatment for moderate and severe pain [12]. A study in a hospital in Brazil reported the average pain severity of patients in the medical ward to be 6.6/10 with 68% of the patients having inadequate therapy for their pain [13]. The prevalence of acute pain in some settings in Africa is as high as 91% among inpatients [14] and about 75% among outpatients [15]. The prevalence of inadequately treated pain was 68%  in a teaching hospital in Ethiopia [13] and 66% in a national hospital in Kenya [16].

Low income countries have only 6% of the world’s narcotic analgesics while 89% are used in the western countries [17]. In the case of Uganda, the palliative care burden on the healthcare system is approximately 137,700 patients which includes patients with HIV, cancer, renal disease, respiratory illnesses, neurological disorders, cardiovascular conditions and liver disease. Fifty eight percent of these have no access to pain relief [18]. Hospice Uganda has conducted trainings for nurses and clinical officers on pain management and prescription protocols for narcotics like morphine. However, this landmark progress is challenged by; stock outs of oral morphine in health centers, inability of patients to afford its cost, unsustainability of the training program [19]. Studying pain and its management among patients is crucial in formulating measures to ensure the patients experience pain relief. This study aimed at studying prevalence of relief from acute pain and associated factors in medical ward of Mbarara Regional Referral Hospital (MRRH), South Western Uganda. This will contribute knowledge that can be leveraged when mitigating the problem of inadequate pain therapy and inadequate pain relief.

## Materials and methods

### Design

This was a descriptive cross-sectional study.

### Setting

This study was conducted in Mbarara Regional Referral Hospital. The participants in the study were enrolled from among the patients admitted on medical ward. Both males and females were included. Mbarara Regional Referral Hospital, commonly known as Mbarara Hospital, is a public referral hospital and a teaching hospital for the Medical School of Mbarara University of Science and Technology, South Western Uganda. The hospital was founded in 1940 and it currently has a bed capacity of 350. It is located in Mbarara town in South Western Uganda along Mbarara Kabale road 266km from Kampala Capital City. The hospital serves a population of over four (4) million people in its catchment area comprising 15 districts of South Western Uganda (Mbarara, Mbarara City, Sheema, Bushenyi, Rwampara, Lyantonde, Rakai, Ntungamo, Kazo, Kiruhura, Ibanda, Buhweju, Rubirizi, Mitooma, Isingiro districts), and the neighboring countries including Burundi, Democratic Republic of Congo, Rwanda, and Tanzania. The medical ward has 50 beds and approximately 300 patients are admitted each month. It has a side laboratory that provides rapid testing as well as diagnostic and monitoring tests at the point of care.

### Characteristics of participants

Patients who were 18 years and above, diagnosed with acute pain and admitted in the medical ward during the study period were included in this study. Those who experienced only neuropathic pain, were unconscious, discharged or died before follow up pain assessment were excluded.

### Sample size determination

A study done in Pakistan reported a 76% level of satisfaction to management of acute pain among patients admitted on a medical ward [20]. This rate was adopted for the calculation of the sample size of this study considering that both settings are teaching and referral hospitals in low and middle-income countries. There is limited data from sub-Saharan Africa published on relief of acute pain specifically among patients on medical wards.


$$n=\left(z²pq\right)/e²$$

Where;

n = sample size

p= proportion (76%)

q= (1-p)

e= margin error (5%)

z = 1.96 confidence level

n= (1.96²×0.76×0.0.24)/0.05²

n=280 patients with acute pain.

After adding 10% contingency, 308 patients were enrolled.

### Sampling technique

Participants were enrolled by consecutive sampling every day. Each participant’s pain was assessed at the point of enrollment into the study (day one) and follow up assessment was done on day two.

### Data collection and procedures

Enrollment of eligible participants started on 24^th^ October 2022 and ended on 15^th^ February 2023. All participants gave written informed consent before recruitment. Acute pain was diagnosed by asking the patient if they were experiencing pain which had lasted not more than three months. If the patient admitted to having such pain the research physician performed a physical examination at the site of pain to confirm the patient’s report.

A participant was enrolled by the study nurse after a diagnosis of acute pain had been made by the physician. The severity of pain for every enrolled participant was assessed on day one and another assessment was done on day two after enrollment. Severity of pain was determined using the Brief Pain Inventory (BPI) which rates pain on a scale of 0 to 10 where 0 means no pain and 10 means the worst pain the patient has ever experienced. The worst pain over 24hours reported by the patient was considered for pain grading as follows; 0 for “no pain”, 1 to 3 for “mild pain”, 4 to 6 for “moderate pain”, and 7 to 10 for “severe pain”.

### Adequacy of therapy for acute pain

Adequate drug therapy for acute pain was assessed using the pain management index (PMI). The patients’ pain grades i.e., no pain, mild, moderate and severe acute pain were labelled as 0, 1, 2, and 3, respectively. Prescribed analgesic drug was scored as “0” for no prescribed analgesic drug, “1” for non-opioid drug e.g., paracetamol, NSAIDS, “2” for weak opioids e.g., codeine, tramadol and “3” for strong opioids e.g., morphine, fentanyl. The PMI value for each patient was then computed by subtracting the patient’s pain grade from the score of analgesic drug class. The PMI values ranged from −3 for a patient with severe pain, but no prescribed analgesic drug to +3 for a patient reporting no pain but having been prescribed a strong opioid. Negative PMI values were considered to indicate inadequate drug therapy for acute pain while positive PMI values or zero were considered an indicator of adequate drug therapy for acute pain [21].

### Relief from acute pain

Relief from acute pain which was the primary outcome variable of this study was considered a change in pain grade from severe to mild or moderate to mild or mild to no pain as obtained by comparing pain grade on day one after enrollment from follow up pain grade on day two.

### Factors associated with relief from acute pain

A checklist was used to collect data on factors associated with relief from acute pain. Data was collected both by interviewing the patient and reviewing patient files. The checklist was designed by categorizing the factors into; patient related associated factors like age, gender, occupation category, address, disease related factors like nature of illness, presence of comorbidities, duration of illness, nature of treatment, presence of renal or liver dysfunction, drug related factors like prescribed analgesic, number of other prescribed drugs, administration status of the analgesic, reason why prescribed analgesic was not administered, pain related factors like severity of pain, duration of pain, cause of pain, description of pain.

### Data analysis

Data was entered into Microsoft excel 2010 and imported into SPSS software version 20 for analysis. Prevalence of adequate drug therapy for acute pain and proportion of relief from acute pain were obtained using descriptive statistics.

Association between relief from acute pain and the independent variables were analyzed by performing logistic regression. The variables with p-values below 0.25 were considered for multivariate analysis after which all variables that had p-values below 0.05 at 95% confidence interval were considered to have statistically significant association with relief from acute pain.

### Statement of ethics compliance

The Mbarara University of Science and Technology Research Ethics Committee approved this study with a reference number MUST-2022-568. The study was conducted in compliance with the Declaration of Helsinki. All participants gave written informed consent before data was collected from them.

## Results

### Demographic characteristics

A total of 280 patients had their pain assessed on day of enrollment (day one) and follow up assessment was done on day two. They had a median (IQR) age of 46.0 (34.3-60.0). About half (139, 49.6%) were males, and most (214, 76.9%) had occupations classified by International Standard Classification of Occupations 2008 (ISCO 08) as skill level 1. Over a half (167, 59.6%) had comorbidities in addition to their primary diagnosis, 105(37.6%) had at least one of renal or liver dysfunction and 128(45.7%) were prescribed more than one drug. The causes of acute pain afflicting the study participants were categorized using the International Classification of Diseases 2011 (ICD 11). Majority of participants got classified into; ‘infectious and parasitic diseases’ 61(22.1%) followed by ‘diseases of the circulatory system’ 61(22.1%). Most of the participants reported only one site of pain (213, 76.1%) and duration of pain of not more than one month (230, 82.1%) (Table 1).

**Table 1:** Sociodemographic, clinical and drug characteristics of patients with acute pain admitted in medical ward of MRRH, South Western Uganda.

Variable	Category	Count	Percentage (%)
Age category (Median [IQR]=46.0 (34.3-60.0))	<35	69	24.6
	35-59	140	50.0
	>=60	71	25.4
Gender	Male	139	49.6
	Female	141	50.4
Occupation skill level (ISCO 08)	1	214	76.4
	2	57	20.4
	3	9	3.2
Nature of disease	Infectious	119	42.5
	Non-infectious	161	57.5
Comorbidity	No	113	40.4
	Yes	167	59.6
Organ dysfunction	No	174	62.4
	Yes	105	37.6
Nature of treatment	Non-invasive	39	13.9
	Invasive	241	86.1
Number of other drugs	<2	152	54.3
	≥2	128	45.7
Disease classification (ICD-11)	Diseases of blood or blood forming organs	18	6.4
	Diseases of the circulatory system	62	22.1
	Diseases of the digestive system	61	21.8
	Diseases of the nervous system	9	3.2
	Diseases of the respiratory system	11	3.9
	Infectious/parasitic diseases	62	22.1
	Neoplasms	27	9.6
	Others*	30	10.7
Duration of pain in weeks	<1	128	45.7
	1-4	102	36.4
	>4	50	17.9
Number of pain sites	One	213	76.1
	More than one	67	23.9
Pain relief	No	185	66.1
	Yes	95	33.9

*Chronic kidney disease, diabetes mellitus, eczema, rheumatoid arthritis

### What is the prevalence of adequate drug therapy for acute pain among patients admitted at medical ward of MRRH?

**Fig 1:**
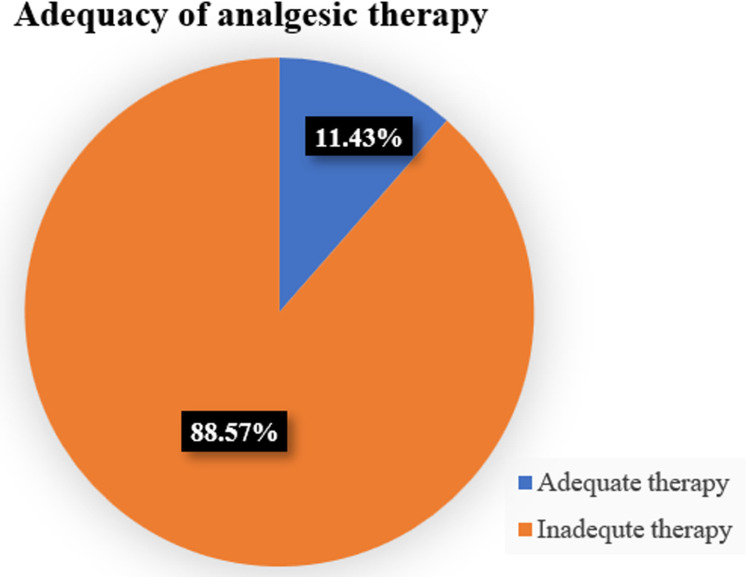
Adequacy of acute pain therapy for patients admitted in medical ward of MRRH, South Western Uganda.

Only over a tenth (32, 11.43%) of the participants had adequate analgesic therapy for their acute pain while a vast majority, (248, 88.57%) of the participants had inadequate therapy (Fig 1).

Out of 193 patients with severe pain, only 21(10.9%) received score 3 analgesics whereas the majority 107(55.4%) had no prescription at all for their pain (Table 2).

**Table 2:** Derivation and summary of PMI grades for pain severity and prescribed analgesic drug scores.

PMI pain grade	Score of prescribed analgesic drug (%)				
	0	1	2	3	Total (%)
1*(Mild*)	**2** *	1*	0	0	3(1.1%)
2*(Moderate)*	**57(67.9%)**	**18(21.4%)**	4(4.8%)	5(6.0%)	84(30.0%)
3*(Severe)*	**107(55.4%)**	**41(21.2%)**	**24(12.4%)**	21(10.9%)	193(68.9%)
% Within pain grade	166(59.3%)	60(21.4%)	28(10.0%)	26(9.3%)	280(100%)

* Percentage not appropriate because of low denominator; Italics: Categorization of pain as mild, moderate and severe is based on participants’ BPI ratings; **Bold**: Counts and percentages of participants with inadequate pain therapy within various PMI pain grades.

**Fig 2:**
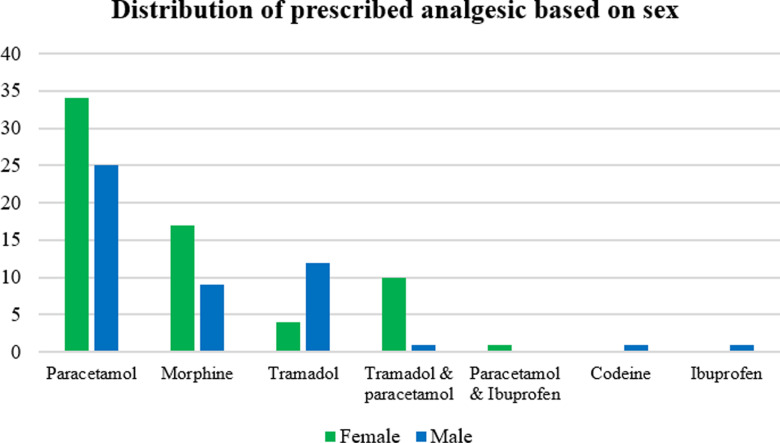
Distribution of prescribed analgesic drugs based on gender for patients with acute pain admitted in medical ward of MRRH, South Western Uganda.

Out of 115 analgesic drug prescriptions, more than half (66, 57.4%) were written for females while only (49, 42.6%) were for males (Fig 2). Female patients were written for more prescriptions for paracetamol (34, 57.6%), morphine (17, 65.4%) and tramadol (10, 90.9%).

### What is the proportion of patients with relief from acute pain among in medical ward of Mbarara Regional Referral Hospital?

**Fig 3:**
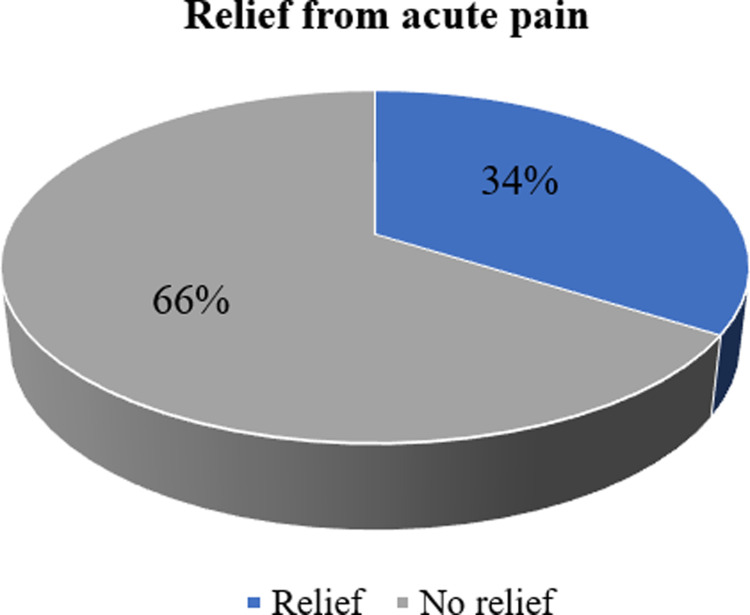
Percentages of patients who experienced relief from acute pain after four days of follow up.

Only a third (95, 34%) of the patients experienced pain relief while two thirds (185, 66%) did not (Fig 3).

### What factors are associated with relief from acute pain in medical ward of Mbarara Regional Referral Hospital?

A total of nine variables were considered in univariate analysis from which six qualified for multivariate analysis. Only gender was found to be significantly associated with pain relief (aOR=1.86; 1.11-3.10 at 95% C.I; p-value=0.018). Female patients showed about 1.86 times odds of getting relief from acute pain compared to males (Table 3)

**Table 3:** Univariate and multivariate logistic regression results for factors associated with relief from acute pain.

Variables	Categories	Pain relief		cOR (95% CI)	P value	aOR (95% CI)	P value
		No	Yes				
Gender	Male	101(72.7)	38(27.3)	1			
	Female	84(59.6)	57(40.4)	1.80(1.09-2.98)	**0.021**	1.86(1.11-3.10)	**0.018**
Age	<35	43(62.3)	26(37.7)	1			
	35-59	102(72.9)	38(27.1)	0.62(0.33-1.14)	**0.122**	0.60(0.32-1.13)	0.112
	≥60	40(56.3)	31(43.7)	1.28(0.65-2.52)	0.472	1.19(0.60-2.36)	0.626
Occupation (Skill level)	1	141(65.9)	73(34.1)	4.14(0.51-33.76)	**0.184**	2.40(0.28-20.47)	0.425
	2	36(63.2)	21(36.8)	4.67(0.55-39.96)	**0.160**	3.14(0.35-28.10)	0.307
	3	8(88.9)	1(11.1)	1			
Nature of illness	Infectious	79(66.4)	40(33.6)	0.98(0.59-1.61)	0.924		
	Non infectious	106(65.8)	55(34.2)	1			
Comorbidity	No	73(64.6)	40(35.4)	1.12(0.68-1.85)	0.669		
	Yes	112(67.1)	55(32.9)	1			
Organ dysfunction	No	111(63.8)	63(32.2)	1.38(0.82-2.32)	**0.229**	1.34(0.78-2.30)	0.297
	Yes	74(70.5)	31(29.5)	1			
Nature of treatment	Non-Invasive	29(74.4)	10(23.6)	0.63(0.29-1.36)	**0.242**	0.76(0.34-1.71)	0.511
	Invasive	156(64.7)	85(35.3)	1			
Duration of pain	<1week	82(64.1)	46(35.9)	1.31(0.65-2.65)	0.454		
	1-4 weeks	68(66.7)	34(33.3)	1.17(0.56-2.43)	0.680		
	>4weeks	35(70.0)	15(30.0)	1			
ICD 11	Diseases of blood or blood forming organs	11(61.1)	7(38.9)	1.82(0.51-6.54)	0.360	1.80(0.48-6.79)	0.338
	Diseases of the circulatory system	41(66.1)	21(33.9)	1.46(0.53-4.01)	0.459	1.66(0.57-4.80)	0.354
	Diseases of the digestive system	37(60.7)	24(39.3)	1.85(0.68-5.05)	**0.228**	2.03(0.70-5.88)	0.190
	Diseases of the nervous system	5(55.6)	4(44.4)	2.29(0.46-11.00)	0.303	3.98(0.72-22.08)	0.114
	Diseases of the respiratory system	7(63.6)	4(36.4)	1.63(0.36-7.32)	0.522	1.45(0.30-7.13)	0.648
	Infectious/parasitic diseases	41(66.1)	21(33.9)	1.46(0.53-4.01)	0.459	1.71(0.59-4.95)	0.321
	Neoplasms	20(74.1)	7(25.9)	1			
	Others*	23(76.7)	7(23.3)	0.87(0.26-2.91)	0.820	1.03 (0.29-3.68)	0.965
No. of pain sites	1	134(62.9)	79(37.1)	1.88(1.00-3.52)	**0.048**	1.74(0.92-3.32)	0.090
	>1	51(76.1)	45(23.9)	1			
No. of other drugs	≤2	102(67.1)	50(32.9)	0.90(0.55-1.49)	0.691		
	>2	83(64.8)	45(35.2)	1			

* Chronic kidney disease, diabetes mellitus, eczema, rheumatoid arthritis; **Bold**: p values less than 0.25 at level of univariate analysis. These variables are the ones considered for multivariate analysis; cOR: crude Odds Ratios, aOR: adjusted Odds Ratios; ICD-11: International Classification of Diseases 2011; ISCO-08: International Standard Classification of Occupations 2008

## Discussion

Out of the 280 participants studied, only 115 (41.1%) were prescribed for analgesics while only 32 (11.43%) received adequate analgesic therapy. In the current study adequacy of analgesic therapy was significantly lower compared to 79% observed in a referral hospital in Kenya [16], 78% observed among adult patients in Iceland [22] and 32% reported in a university hospital in the city of Sao Paolo [23]. Higher adequacy of analgesic therapy in the previous studies may be attributed to the use of pain severity scales during assessment of pain unlike in the setting of the current study where pain is largely empirically managed. Low adequacy of pain therapy observed in the current study could be due to; limited access to opioids [24], tight legal restrictions on opioid prescription [25] and poor documentation of outcomes of pain assessment and therapy [26].

Pain in medical wards is generally given limited attention and this hinders the achievement of relief. This is supported by the findings from a retrospective study at a tertiary hospital in Portugal which demonstrated that pain among patients in medical ward was under diagnosed and under managed [27]. Review of patient files in the current study showed that none of the studied patients had their pain assessment documented by the attending medical team. This has been associated with inadequate pain therapy as demonstrated in a prospective non interventional study done among cancer patients in India [26].

Out of 39 patients whose prescribed analgesic drug was not administered, majority (16, 41.0%) reported that they could not afford to buy. Uganda is a low-income country. Low income status is associated with drug stock outs and shortage of health workers [28]. Some patients may already be financially constrained by other costs related to their illnesses because of poor socioeconomic status. These negatively affect efforts to adequately manage pain.

It has also been observed that there is a fear to prescribe opioids among health workers of Uganda as morphine has been erroneously associated with high risk of dependency [29]. Dispensing of strong opioids like morphine and pethidine involve relatively stringent regulatory and logistical procedures [24,30]. These may have discouraged clinicians from prescribing strong opioids for patients who might have needed them. This could explain why in spite of 193 patients reporting severe pain, the clinicians prescribed morphine for only 21(10.9%) hence resulting into low adequacy of therapy for acute pain.

Two days after diagnosis of acute pain, only a third (95, 34%) of the 280 patients had experienced relief from acute pain. This is comparable to results from a retrospective study from 40 hospitals in Italy which reported that only 19% of the patients on analgesic drug therapy enjoyed good pain control [31]. A study on pain management in inpatient wards of a university hospital in Brazil demonstrated that patients in medical ward stayed in pain longer compared to those admitted to surgical units [32]. These findings build a case for the global need for health workers to pay more attention to acute pain in medical wards. The low prevalence of relief from acute pain observed in the current study is unlike what was reported from Aga Khan University Hospital Karachi, Pakistan where 76% of patients from medical ward were satisfied with their pain management [20]. However, it is important to note that patient satisfaction with pain management may not be necessarily translate to pain relief.

This low prevalence of relief from acute pain is in correlation with low prevalence of adequacy of acute pain therapy demonstrated in this study. It was observed that more than a half of the studied patients (107, 55.4%) had severe pain but were not prescribed any analgesic drug. Paracetamol was the most prescribed analgesic despite majority (193, 68.9%) of the patients reporting severe pain. This demonstrates a failure by the clinicians to accurately diagnose the type and severity of pain as per the pain guidelines like the Uganda Clinical Guidelines (UCG). In Uganda adherence to UCG among health workers in hospitals was observed to be remarkably low at 30% [33]. In addition, the pathophysiology of pain may vary from patient to patient and therefore individualized treatment is key for efficacious and safe therapy [34]. This warrants elaborate assessment of patients’ pain on a case by case basis with effective communication skills in order to achieve accurate diagnosis and treatment [35].

On performing multivariate analysis female gender was 1.86 times associated with relief from acute pain as compared to male gender. It was observed that out of 115 precriptions of analgesics written by the attending clinicians more than half (66, 57.4%) were for female patients. A study done in Dallas, Texas demonstrated that female patients were more likely to complain about their pain and also overscore pain severity compared to their male counterparts [36]. As a result clinicians may pay more attention to female patients’ pain [37] which could explain why in the current study more opioids (31 out of 54, 57.4%) were prescribed for female patients compared to males. Females are also more likely to use OTC analgesics than males as demonstrated by a study in Norway [38].

According to the WHO pain is a vital sign that must be assessed for every patient. There is need for health workers to be trained on the various pain assessment methods. This could enhance health worker adherence to pain assessment protocols. There is a scarcity of studies on pain among patients admitted on medical wards. This has led to pain among medical patients being generally ignored. More studies like the current one will unearth novel evidence that will attract attention of researchers and health workers to this understudied setting and population.

### Study limitation and strength

#### Limitation.

The PMI has no numerical representation for adjuvant analgesics like amitriptyline and carbamazepine therefore their role in treatment of pain is not accounted for. This could have been a confounder in this study.

#### Strength.

In the current study all independent variables included in the analysis could be objectively verified and therefore bias was minimized. This ensured reliability of the study findings.

## Conclusion

Acute pain has largely been associated with patients admitted in surgical, emergency, obstetrics and gynecology wards. On the other hand, it has received limited attention in medical wards. There is a conspicuous scarcity of published studies on acute pain in medical wards. This study demonstrated the gap in management and relief of acute pain in medical wards. Nevertheless, we believe that acknowledging acute pain as a health problem among patients admitted in medical wards is important. In the current study prevalence of adequacy of analgesic drug therapy for acute pain was low. This was possibly because there was low adherence to available treatment guidelines like the Uganda Clinical Guidelines with regard to pain management. The proportion of patients with relief from acute pain was also low. This corresponds with the low prevalence of adequacy of analgesic drug therapy. Female gender was found to be significantly associated with relief from acute pain most likely because female patients were generally prescribed more analgesic drugs compared to the males.
